# Effects of *apoC1* genotypes on the hormonal levels, metabolic profile and PAF-AH activity in Chinese women with polycystic ovary syndrome

**DOI:** 10.1186/s12944-018-0725-5

**Published:** 2018-04-10

**Authors:** Renjiao Zhang, Qingqing Liu, Hongwei Liu, Huai Bai, Yujin Zhang, Linbo Guan, Ping Fan

**Affiliations:** 1 Laboratory of Genetic Disease and Perinatal Medicine, Key Laboratory of Birth Defects and Related Diseases of Women and Children, Ministry of Education, West China Second University Hospital, Sichuan University, Chengdu, Sichuan 610041 People’s Republic of China; 2 Department of Obstetrics and Gynecology, West China Second University Hospital, Sichuan University, Chengdu, Sichuan 610041 People’s Republic of China

**Keywords:** apoC1, Gene polymorphism, Lipoprotein, PAF-AH activity, apoE, Polycystic ovary syndrome

## Abstract

**Background:**

Elevated serum levels of apolipoprotein (apo) C1 may be an early protein marker of metabolic abnormality in women with polycystic ovary syndrome (PCOS). It is not clear, however, whether there are any relationships between the *apoC1 rs4420638A/G* and *-317deletion (H1)/insertion (H2)* polymorphisms and PCOS. We investigated the relationship between these two variants and the risk of PCOS, evaluated the genotypic effects on clinical, hormonal and metabolic indexes and plasma platelet-activating factor acetylhydrolase (PAF-AH) activity, and defined the association of *apoC1* gene variants with *apoE ε2/ε3/ε4* polymorphisms.

**Methods:**

This is a cross-sectional study of 877 women with PCOS and 761 controls. The *apoC1 rs4420638A/G* genotype was determined by a Taqman real-time PCR allelic discrimination assay. The *apoC1–317H1/H2* and *apoE ε2/ε3/ε4* genotypes were measured using PCR and restriction fragment length polymorphism analysis. The clinical, hormonal and metabolic parameters and PAF-AH activity were measured.

**Results:**

The frequencies of *apoC1 rs4420638A/G* and *-317H1/H2* genotypes and alleles were similar between PCOS and control groups (*P* > 0.05). However, the *rs4420638 G* allele was related to increased serum luteinizing hormone, cholesterol and apoB levels, and the ratio of apoB to apoA1 (*P* < 0.05), and the *-317H1H1* genotype was associated with a higher acne grade score and a higher ratio of apoB-PAF-AH to H-PAF-AH activity (*P* < 0.05) in patients with PCOS. We also demonstrated that the *apoC1 rs4420638A/G* and *-317H1/H2* gene variants existed in moderate to reasonably high linkage disequilibrium with *apoE ε2/ε3/ε4* polymorphisms in Chinese women.

**Conclusion:**

The *apoC1 rs4420638A/G* and *-317H1/H2* gene variants might be involved in endocrine abnormalities of reproductive axis, metabolic abnormalities and chronic inflammation in PCOS, although no association was observed between the apoC1 genetic variants and the risk of PCOS in Chinese women.

## Background

Polycystic ovary syndrome (PCOS) is a common heterogeneous female endocrine metabolic disorder affecting 4–18% of reproductive-aged women [1, 2]. In addition to reproductive disorders, PCOS is often associated with long-term cardiovascular health risks, including obesity, visceral obesity, insulin resistance, hyperinsulinemia, dyslipidemia, increased oxidative stress, chronic low-grade inflammation, endothelial dysfunction, vascular preclinical abnormalities, elevated risks of metabolic syndrome, impaired glucose tolerance, type 2 diabetes, and future cardiovascular diseases [2–7]. The etiology of PCOS remains obscure, but studies have suggested that PCOS appears to have a complex, multifactorial etiology resulting from the interactions between genetic, environmental and intrauterine factors [8, 9].

Apolipoprotein (apo) C1 is a constituent of high-density lipoprotein (HDL) and triglyceride-rich lipoproteins and plays an important role in the regulation of lipoprotein metabolism and serum lipid levels. ApoC1 affects catabolism and clearance of apoB-containing lipoproteins by inhibiting lipoprotein lipase (LPL), liver-specific low density lipoprotein (LDL) receptor (LDLR) and LDLR-related protein (LRP) [10–12]. This elevates serum levels of cholesterol and triglycerides (TG) in human apoC1–transgenic mice [10–12].

The human *apoE–apoC1–apoC4–apoC2* gene cluster is located on chromosome 19. Several genome-wide association studies have demonstrated that the *rs4420638 A → G* variant of the *apoC1* gene, located in the non-coding region, is associated with increased serum total cholesterol (TC) and LDL-C levels [13–15], plasma glucose concentration [16] and platelet-activating factor acetylhydrolase (PAF-AH) activity [17]. Furthermore, the *rs4420638 A → G* variant is associated with elevated risks of metabolic syndrome, type 2 diabetes, and coronary heart disease [14, 16, 17]. However, the mechanism by which the *apoC*1 *rs4420638A/G* variant influences plasma lipid levels and increases the risks of diseases is unclear. A 4-bp *CGTT* deletion (*H1*)/insertion (*H2*) polymorphism (rs11568822) at the − 317 site in the *apoC*1 promoter region has been reported to affect apoC1 gene expression [18, 19] and is related to the risks of late-onset Alzheimer’s disease [20] and type III hyperlipoproteinemia [21]. In addition, the *rs4420638A/G* and *-317H1/H2* polymorphisms of the *apoC1* gene have been reported to exist in linkage disequilibrium with *apoE ε2/ε3/ε4* polymorphisms but exhibit a different degree of linkage disequilibrium in populations of different races [19, 22].

The elevated serum apoC1 levels in patients with PCOS, even PCOS patients with normal weight or normolipemic indexes, suggest that apoC1 may be an early protein marker of metabolic abnormality in these patients [23]. However, to date, little information is available regarding the possible connection between the *apoC1 rs4420638A/G* and *-317H1/H2* polymorphisms and PCOS and the degree of linkage disequilibrium between these variants and *apoE ε2/ε3/ε4* polymorphisms in Chinese women. In the present study, we investigated the relationship between the *rs4420638A/G* and *-317H1/H2* polymorphisms of the *apoC1* gene and the risk of PCOS, evaluated the effects of the genotypes on clinical and metabolic indexes and PAF-AH activity and defined the association of *apoC1* gene variations with *apoE* allele status in southwest Chinese women with or without PCOS using relatively large sample sizes.

## Methods

### Study subjects

Women with or without PCOS aged 17 to 40 years were recruited from 2006 to 2015 from the Outpatient Clinic of Reproductive Endocrinology at West China Second University Hospital. For the genetic association study of *apoC1* and *apoE* polymorphisms, we included 877 patients and 761 controls. For association studies between *apoC1* genotypes and hormonal levels, metabolic parameters, and PAF-AH activity, the subjects were excluded if they met one of the following criteria: [i] taking medication known to affect the metabolism of carbohydrates, lipids, or hormones within 3 months before the study; [ii] being pregnant or in the luteal phase; and [iii] smoking. Finally, 575 patients and 506 controls were included in the metabolism-related study. In addition, PAF-AH activity was measured in 281 patients and 277 controls of the metabolic analysis groups.

Each patient with PCOS met the revised 2003 Rotterdam ESHRE/ASRM consensus criteria [24]. Oligo-ovulation or anovulation (OA) was assessed as oligomenorrhea (i.e., fewer than eight cycles per year). Clinical or biochemical hyperandrogenism (HA) was assessed by three measures: hirsutism with a modified Ferriman–Gallwey (F-G) score of more than 6, clinical presence of obvious acne and/or total testosterone (TT) level ≥ 2.60 nmol/L [3, 4, 25]. Polycystic ovaries (PCOs) were confirmed if there were 12 or more follicles in each ovary measuring 2 to 9 mm in diameter and/or increased ovarian volume (> 10 mL) by ultrasonic examination. The diagnosis of PCOS was based on a patient having two of these three findings with HA as an essential condition for women aged < 20 years [2] and exclusion of other etiologies such as androgen-secreting tumors, congenital adrenal hyperplasias, and Cushing syndrome. All the controls were clinically healthy women who had regular menstrual cycles (between 21 and 35 days), exhibited normal circulating androgen levels, did not show hirsutism or obvious acne on physical examination, and had normal ovarian morphology as determined by ultrasound.

None of the subjects had clinically evident acute or chronic diseases, such as infection, tumors, cardiovascular disease, thyroid dysfunction, endometriosis, hyperprolactinemia, hypogonadotropic hypogonadism or premature ovarian insufficiency.

Clinical and anthropometrical parameters, including waist circumference, waist-to-hip ratio, body mass index (BMI, kg/m^2^), systolic and diastolic blood pressure (SBP and DBP), the degree of hirsutism and acne, and ultrasound ovarian volume [26] were measured or assessed in all subjects.

Blood samples were obtained in the morning after overnight fasting on day 3–10 of the menstrual cycle from regularly menstruating women or at random from women with OA. Samples were placed on ice immediately and centrifuged at 1500×g for 15 min at 4 °C within 2 h. Plasma and serum samples were stored at − 80 °C. Blood cells were stored at 4 °C.

### Genotype analysis

Genomic DNA was isolated from peripheral blood leukocytes of the subjects [27, 28]. For the *apoC1 rs4420638* genotype, a 101-bp fragment was amplified and detected by a Taqman real-time PCR allelic discrimination assay using the forward primer, 5’-TCAGCCTAGCAATGTCACTATGC-3′, the reverse primer, 5’-GTCTGCCTCAAAACAGAAACAAAA-3′, the wild-type probe, HEX-CTTTTCCTaGTGTGGTCTA-TAMRA and the mutant probe, FAM-CACTTTTCCTgGTGTGGT-TAMRA. The 151-bp wild-type (A) or mutant (G) DNA fragments of the *apoC1* gene were used as positive controls. The primers, probes and positive controls were designed and synthesized by the Genecore Biotech Co. Ltd., Shanghai, China. For the *-317H1/H2* genotype, the 221-bp (*H1, CGTT* deletion) and 225-bp (*H2, CGTT* insertion) fragments were amplified using the forward primer: 5’-TTTGAGCTCGGCTCTTGAGACAGGAA-3′ and the reverse primer 5’-GGTCCCGGGCACTTCCCTTAGCCCCA-3′ [29]. The *-317H1/H2* PCR products were digested with HpaI (Thermo) and analyzed by electrophoresis on a 3.0% agarose gel and visualized by staining with Genecolour fluorescent dye. The enzyme digestion resulted in 159- and 66-bp fragments for the *-317H2* allele and a non-digested 221-bp fragment for the *-317H1* allele. *ApoE ε2/ε3/ε4* polymorphisms (rs429358 and rs7412) were measured as described previously [30]. For quality control, more than 30% of DNA samples were genotyped again by a different operator.

### Analysis of PAF-AH activity, hormonal markers and metabolic markers

Serum luteinizing hormone (LH), follicle stimulating hormone (FSH), estradiol (E_2_), TT, triglyceride (TG), total cholesterol (TC), HDL-cholesterol (HDL-C), LDL-cholesterol (LDL-C), apoA1, apoB, plasma glucose and insulin concentrations, plasma PAF-AH, apoB-containing lipoprotein-associated PAF-AH (apoB-PAF-AH) and HDL-associated PAF-AH (H-PAF-AH) activity were measured as previously described [4, 31, 32]. Additionally, the atherogenic index (AI) and the homeostatic model assessment of insulin resistance (HOMA index) were assessed as described previously [4, 31]. The intra- and inter-assay coefficients of variation for all measurements were less than 5 and 10%, respectively.

### Statistical analysis

Data were presented as the mean ± standard deviation (SD). Differences in variables were evaluated by an independent sample t-test between PCOS and control subjects. Variables with asymmetric distribution were evaluated by a Mann-Whitney U test. A chi-square analysis was used to determine allele or genotype frequencies and to test deviations in the genotype distribution from Hardy-Weinberg equilibrium between patients and controls. An analysis of covariance (ANCOVA) or a two way analysis of variance (ANOVA) was used to estimate the differences in clinical parameters, hormonal levels, metabolic profile and PAF-AH activity between the two groups or genotype subgroups after correction for differences in age and BMI. A *P*-value of < 0.05 was considered to be statistically significant. All statistical analyses were performed using the Statistical Program for Social Sciences (SPSS) 13.0 for Windows (Chicago, IL, USA).

The analysis of linkage disequilibrium between different genetic polymorphic loci was performed by the SHEsis online software at http://analysis.bio-x.cn/myAnalysis.php.

## Results

### Clinical and biochemical characteristics of the study population

Because mean age and BMI were different between the PCOS and the control groups (Table 1), differences that could bias comparisons of other clinical parameters, the hormonal and metabolic indexes and PAF-AH activity between the two groups were adjusted for the difference in age and BMI in the following analysis.

**Table 1:** Clinical characteristics of PCOS patients and controls

	Controls (*n* = 761)	PCOS (*n* = 877)	*P*	*P* ^*a*^
Age (years)	28.19 ± 4.14	24.66 ± 3.95	< 0.001	
BMI (kg/m^2^)	21.18 ± 2.95	22.79 ± 4.04	< 0.001	
Waist circumference (cm)	73.63 ± 8.23	78.74 ± 11.17	< 0.001	< 0.001
Waist-to-hip ratio	0.82 ± 0.06	0.85 ± 0.07	< 0.001	< 0.001
F-G score	0.22 ± 0.71	1.68 ± 2.03	< 0.001	< 0.001
Acne grade score	0.09 ± 0.29	0.60 ± 0.90	< 0.001	< 0.001
SBP (mmHg)	113.20 ± 11.72	114.09 ± 10.62	0.110	0.743
DBP (mmHg)	73.87 ± 9.25	75.51 ± 8.92	< 0.001	0.089
Ovarian volume (ml)	7.53 ± 2.84	9.90 ± 4.09	< 0.001	< 0.001

Values are presented as the mean ± SD

*P*^*a*^ All comparisons were corrected for differences in age and BMI between the two groups except the parameters of age and BMI

As shown in Table 1, BMI, waist circumference, waist-to-hip ratio, F-G score, acne grade score, and average ovarian volume were significantly increased, and age was significantly decreased in the PCOS group compared with the control group.

TT and LH levels, the ratio of LH to FSH, fasting insulin concentration, HOMA index, TG, TC, LDL-C, non-HDL-C and apoB levels, AI, the ratio of apoB to apoA1, and the ratio of apoB-PAF-AH to H-PAF-AH were significantly increased, whereas FSH and HDL-C levels and H-PAF-AH activity were significantly reduced in the PCOS group compared with the control group (Table 2).

**Table 2:** Hormonal levels, metabolic profile and PAF-AH activity in PCOS patients and controls

	Controls (*n* = 506)	PCOS (*n* = 575)	*P*	*P* ^*a*^
Age (years)	27.96 ± 4.19	24.66 ± 3.96	< 0.001	
BMI (kg/m^2^)	21.10 ± 2.91	23.10 ± 4.26	< 0.001	
Hormonal levels				
E_2_ (pmol/L)	333.95 ± 351.36	284.06 ± 271.26	0.014	0.373
TT (nmol/L)	1.54 ± 0.54	2.42 ± 0.77	< 0.001	< 0.001
LH (IU/L)	8.72 ± 11.33	13.89 ± 10.91	< 0.001	< 0.001
FSH (IU/L)	6.67 ± 2.98	5.99 ± 2.23	< 0.001	0.015
LH/FSH	1.30 ± 1.32	2.35 ± 1.26	< 0.001	< 0.001
Metabolic profile				
Fasting Ins (pmol/L)	66.98 ± 36.74	106.08 ± 74.24	< 0.001	< 0.001
Fasting Glu (mmol/L)	5.30 ± 0.73	5.37 ± 0.77	0.110	0.515
HOMA-IR	2.34 ± 1.90	3.79 ± 3.19	< 0.001	0.006
TG (mmol/L)	1.04 ± 0.90	1.42 ± 1.36	< 0.001	< 0.001
TC (mmol/L)	4.25 ± 0.70	4.41 ± 0.81	< 0.001	< 0.001
HDL-C (mmol/L)	1.51 ± 0.32	1.38 ± 0.35	< 0.001	0.006
LDL-C (mmol/L)	2.35 ± 0.61	2.55 ± 0.76	< 0.001	< 0.001
non-HDL-C (mmol/L)	2.73 ± 0.64	3.02 ± 0.81	< 0.001	< 0.001
AI	1.90 ± 0.68	2.37 ± 1.02	< 0.001	< 0.001
ApoA1 (g/L)	1.45 ± 0.21	1.42 ± 0.21	0.005	0.725
ApoB (g/L)	0.75 ± 0.17	0.82 ± 0.20	< 0.001	< 0.001
ApoB/apoA1	0.53 ± 0.14	0.60 ± 0.18	< 0.001	< 0.001
PAF-AH activity^a^				
Plasma PAF-AH (nmol/min/ml)	48.39 ± 10.81	47.44 ± 12.94	0.344	0.722
H-PAF-AH (nmol/min/ml)	5.14 ± 1.51	4.67 ± 1.91	0.001	0.031
ApoB-PAF-AH (nmol/min/ml)	43.25 ± 9.96	42.77 ± 11.89	0.604	0.967
ApoB-PAF-AH/H-PAF-AH	8.86 ± 2.48	10.04 ± 3.89	< 0.001	< 0.001

Values are presented as the mean ± SD

*P*^*a*^ All comparisons of parameters were corrected for differences in age and BMI between the two groups except the parameters of age and BMI

^a^ Control (*n* = 277), PCOS (*n* = 281)

### Distribution of the *apoC1 rs4420638A/G* and *-317H1/H2* genotypes and alleles

Genotypic distributions of *apoC1 rs4420638A/G* and *-317H1/H2* were in Hardy-Weinberg equilibrium in the PCOS and control groups. No significant differences were observed in the frequencies of the *apoC1 rs4420638A/G* and *-317H1/H2* genotypes and alleles between PCOS and control groups (*P* > 0.05, Table 3).

**Table 3:** Frequencies of the *apoC1* genotype and allele in PCOS patients compared with controls

		Controls (n = 761)	PCOS (n = 877)	*X* ^*2*^	*P*
Genotype					
-317	*H1H1*	483 (63.5%)	570 (65.0%)		
	*H1H2*	245 (32.2%)	269 (30.7%)		
	*H2H2*	33 (4.3%)	38 (4.3%)	0.448	0.799
rs4420638	*AA*	578 (76.0%)	683 (77.9%)		
	*AG*	176 (23.1%)	182 (20.8%)		
	*GG*	7 (0.9%)	12 (1.4%)	1.954	0.375
Allele frequency					
-317	*H1*	0.796	0.803		
	*H2*	0.204	0.197	0.297	0.586
rs4420638	*A*	0.875	0.883		
	*G*	0.125	0.117	0.419	0.518

Genotype data are presented as the number (%) of patients or controls

The degree of linkage disequilibrium (LD) among the *apoE ε2/ε3/ε4*, and the *apoC1 rs4420638A/G and -317H1/H2* genetic polymorphisms was analyzed in patients with PCOS and the controls, with all found to be in moderate to reasonably high LD with each other: *apoC1–317H1/H2* loci and *apoE ε2/ε3/ε4* loci (D’ = 0.970, r^2^ = 0.702), *apoC1–317H1/H2* loci and *rs4420638A/G* loci (D’ = 0.910, r^2^ = 0.455), and *apoC1 rs4420638A/G* loci and *apoE ε2/ε3/ε4* loci (D’ = 0.758, r^2^ = 0.397).

### Effects of the *apoC1 rs4420638A/G* and *-317H1/H2* genetic variants on clinical, hormonal and metabolic parameters and PAF-AH activity

Because the sample sizes of the *rs4420638GG* or *-317H2H2* homozygotes were too small, we combined them into the heterozygous subgroups.

As shown in Table 4, compared with *AA* homozygotes, *G* allele carriers (*AG* + *GG*) of the *rs4420638* polymorphism had significantly higher apoB levels and a higher ratio of apoB to apoA1 in patients with PCOS or the controls (*P* < 0.05). Patients with the *G* allele had higher serum LH, TC, LDL-C and non-HDL-C levels (*P* < 0.05), and tended to have a reduced waist-to-hip ratio, DBP and fasting insulin levels (*P* < 0.100) and an increased ratio of LH to FSH (*P* = 0.074) compared with patients with the *AA* genotype. The controls with the *G* allele had higher fasting insulin and glucose concentrations, HOMA indexes, AI, plasma PAF-AH and apoB-PAF-AH activity (*P* < 0.05), and tended to have increased LDL-C and non-HDL-C levels (*P* < 0.080) and decreased HDL-C concentrations (*P* < 0.070) than the controls with the *AA* genotype.

**Table 4:** Clinical characteristics, hormonal levels, metabolic profile and PAF-AH activity of the *apoC1 rs4420638* genotypes in PCOS patients and controls

	Controls		PCOS	
	*AA* (*n* = 379)	*AG + GG* (*n* = 121 + 6)	*AA* (*n* = 435)	*AG + GG* (*n* = 134 + 6)
Age (yr)	28.18 ± 4.10	27.28 ± 4.41^a^	24.68 ± 4.01^a, b^	24.60 ± 3.81^a, b^
BMI (kg/m^2^)	21.17 ± 2.96	20.88 ± 2.74	23.24 ± 4.32^a, b^	22.68 ± 4.03^a, b^
Waist circumference (cm)	73.41 ± 8.21	73.36 ± 8.36	80.13 ± 11.67^a, b^	77.96 ± 11.22^a, b^
Waist-to-hip ratio	0.81 ± 0.06	0.82 ± 0.06	0.86 ± 0.07^a, b^	0.84 ± 0.07^a, b^
F-G score	0.22 ± 0.69	0.26 ± 0.82	1.73 ± 2.07^a, b^	1.71 ± 2.08^a, b^
Acne grade score	0.10 ± 0.30	0.08 ± 0.27	0.66 ± 0.94^a, b^	0.53 ± 0.86^a, b^
SBP (mmHg)	113.42 ± 11.55	112.98 ± 11.84	114.89 ± 10.42^a^	113.78 ± 11.65
DBP (mmHg)	73.97 ± 8.88	73.40 ± 8.75	76.35 ± 9.08^a, b^	74.70 ± 8.99
Ovarian volume (ml)	7.88 ± 2.76	7.47 ± 3.24	10.22 ± 3.92^a, b^	9.95 ± 4.63^a, b^
Hormonal levels				
E_2_ (pmol/L)	322.08 ± 341.61	367.37 ± 376.94	281.07 ± 279.42^b^	293.07 ± 245.85
TT (nmol/L)	1.54 ± 0.54	1.53 ± 0.56	2.41 ± 0.77^a, b^	2.45 ± 0.77^a, b^
LH (IU/L)	8.53 ± 10.38	9.28 ± 13.76	13.24 ± 8.57^a, b^	15.85 ± 15.91^a, b, c^
FSH (IU/L)	6.59 ± 2.84	6.89 ± 3.34	5.95 ± 2.27^a, b^	6.12 ± 2.11^b^
LH/FSH	1.31 ± 1.24	1.29 ± 1.54	2.28 ± 1.18^a, b^	2.58 ± 1.45^a, b^
Metabolic profile				
Fasting Ins (pmol/L)	64.87 ± 32.78	73.28 ± 46.17^a^	109.10 ± 77.82^a, b^	96.60 ± 60.98^a, b^
Fasting Glu (mmol/L)	5.26 ± 0.46	5.42 ± 1.22^a^	5.38 ± 0.71^a^	5.36 ± 0.93^a^
HOMA-IR	2.22 ± 1.22	2.73 ± 3.13^a^	3.89 ± 3.31^a, b^	3.46 ± 2.80^a, b^
TG (mmol/L)	1.02 ± 0.93	1.08 ± 0.76	1.46 ± 1.50^a, b^	1.29 ± 0.73^a^
TC (mmol/L)	4.22 ± 0.71	4.31 ± 0.66	4.35 ± 0.80^a^	4.57 ± 0.82^a, b, c^
HDL-C (mmol/L)	1.52 ± 0.32	1.48 ± 0.29	1.37 ± 0.34^a, b^	1.42 ± 0.36^a, b^
LDL-C (mmol/L)	2.32 ± 0.61	2.44 ± 0.60	2.51 ± 0.75^a^	2.66 ± 0.77^a, b, c^
non-HDL-C (mmol/L)	2.70 ± 0.63	2.83 ± 0.65	2.98 ± 0.81^a, b^	3.14 ± 0.78^a, b, c^
AI	1.87 ± 0.65	2.02 ± 0.74^a^	2.37 ± 1.05^a, b^	2.37 ± 0.91^a, b^
ApoA1 (g/L)	1.46 ± 0.22	1.44 ± 0.18	1.41 ± 0.21^a^	1.43 ± 0.22^a^
ApoB (g/L)	0.74 ± 0.17	0.78 ± 0.17^a^	0.81 ± 0.20^a, b^	0.85 ± 0.20^a, b, c^
ApoB/apoA1	0.52 ± 0.14	0.55 ± 0.16^a^	0.59 ± 0.18^a, b^	0.61 ± 0.19^a, b, c^
PAF-AH activity^d^				
Plasma PAF-AH (nmol/min/ml)	47.80 ± 10.91	50.08 ± 10.39^a^	47.54 ± 12.27^b^	47.05 ± 15.17
H-PAF-AH (nmol/min/ml)	5.09 ± 1.51	5.30 ± 1.52	4.64 ± 1.89^a, b^	4.77 ± 2.01
ApoB-PAF-AH (nmol/min/ml)	42.74 ± 10.09	44.78 ± 9.50^a^	42.91 ± 11.34	42.28 ± 13.74
ApoB-PAF-AH/H-PAF-AH	8.85 ± 2.61	8.88 ± 2.09	10.91 ± 4.17^a, b^	9.48 ± 2.60

Values are presented as the mean ± SD

Comparisons of all parameters were corrected for differences in age and BMI between the two subgroups except the parameters of age and BMI

^a^ *P* < 0.05, compared with *AA* genotype subgroup in controls

^b^*P* < 0.05, compared with *AG + GG* genotype subgroup in controls

^c^*P* < 0.05, compared with *AA* genotype subgroup in PCOS patients

^d^ Controls: *AA* (*n* = 207), *AG + GG* (*n* = 67 + 3); PCOS: *AA* (*n* = 219), *AA + AG* (*n* = 60 + 2)

Compared with patients with the *H1H1* genotype, patients with the *H2* allele (*H1H2* + *H2H2*) in *-317H1/H2* polymorphism had lower acne grade score and the ratio of apoB-PAF-AH to H-PAF-AH (*P* < 0.05). The controls with the *H2* allele had lower TC and LDL-C levels (*P* < 0.05), and tended to have increased waist-to-hip ratio and HOMA index (*P* < 0.09) and reduced non-HDL-C and apoB levels (*P* < 0.09) than the controls with the *H1H1* genotype (Table 5).

**Table 5:** Clinical characteristics, hormonal levels, metabolic profile and PAF-AH activity of the *apoC1–317H1/H2* genotypes in PCOS patients and controls

	Controls		PCOS	
	*H1H1* (*n* = 318)	*H1H2 + H2H2* (*n* = 163 + 25)	*H1H1* (*n* = 370)	*H1H2 + H2H2* (*n* = 177 + 28)
Age (yr)	28.17 ± 4.17	27.56 ± 4.21	24.68 ± 4.03^a, b^	24.63 ± 3.84^a, b^
BMI (kg/m^2^)	21.18 ± 2.95	20.91 ± 2.81	22.94 ± 4.23^a, b^	23.41 ± 4.29^a, b^
Waist circumference (cm)	73.36 ± 7.96	73.35 ± 8.61	79.35 ± 11.36^a, b^	80.06 ± 12.02^a, b^
Waist-to-hip ratio	0.81 ± 0.06	0.82 ± 0.06	0.85 ± 0.07^a, b^	0.85 ± 0.08^a, b^
F-G score	0.25 ± 0.76	0.19 ± 0.68	1.77 ± 2.06^a, b^	1.63 ± 2.10^a, b^
Acne grade score	0.11 ± 0.31	0.07 ± 0.26	0.68 ± 0.96 ^a, b^	0.52 ± 0.86^a, b, c^
SBP (mmHg)	113.32 ± 11.26	113.32 ± 12.22	114.36 ± 10.43^a^	115.08 ± 11.27^a^
DBP (mmHg)	73.81 ± 8.89	73.78 ± 8.76	76.27 ± 9.08^a, b^	75.38 ± 9.06^a^
Ovarian volume (ml)	7.80 ± 2.86	7.71 ± 2.99	10.15 ± 3.99^a, b^	10.15 ± 4.32^a, b^
Hormonal levels				
E_2_ (pmol/L)	324.35 ± 341.91	349.79 ± 366.62	283.87 ± 285.58	284.41 ± 244.71
TT (nmol/L)	1.55 ± 0.53	1.52 ± 0.56	2.41 ± 0.77^a, b^	2.43 ± 0.77^a, b^
LH (IU/L)	8.80 ± 11.33	8.61 ± 11.40	13.65 ± 9.01^a, b^	14.33 ± 13.68^a, b^
FSH (IU/L)	6.64 ± 3.02	6.71 ± 2.92	5.94 ± 1.89^a, b^	6.08 ± 2.74^a, b^
LH/FSH	1.32 ± 1.26	1.28 ± 1.41	2.32 ± 1.20^a, b^	2.42 ± 1.36^a, b^
Metabolic profile				
Fasting Ins (pmol/L)	65.53 ± 34.31	69.27 ± 40.51	105.28 ± 75.66^a, b^	107.54 ± 71.75^a, b^
Fasting Glu (mmol/L)	5.27 ± 0.44	5.35 ± 1.06	5.39 ± 0.84^a^	5.35 ± 0.62
HOMA-IR	2.24 ± 1.27	2.51 ± 2.64	3.80 ± 3.40^a, b^	3.77 ± 2.79^a, b^
TG (mmol/L)	1.03 ± 1.00	1.05 ± 0.68	1.41 ± 1.47^a, b^	1.45 ± 1.13^a, b^
TC (mmol/L)	4.30 ± 0.68	4.15 ± 0.72^a^	4.41 ± 0.81^a, b^	4.39 ± 0.82^b^
HDL-C (mmol/L)	1.52 ± 0.32	1.49 ± 0.31	1.39 ± 0.34^a, b^	1.37 ± 0.35^a, b^
LDL-C (mmol/L)	2.41 ± 0.57	2.26 ± 0.66^a^	2.57 ± 0.76^a, b^	2.51 ± 0.77^a, b^
non-HDL-C (mmol/L)	2.78 ± 0.60	2.66 ± 0.70	3.03 ± 0.82^a, b^	3.02 ± 0.80^a, b^
AI	1.92 ± 0.65	1.88 ± 0.73	2.37 ± 1.03^a, b^	2.37 ± 1.00^a, b^
ApoA1 (g/L)	1.46 ± 0.22	1.44 ± 0.19	1.42 ± 0.21^a^	1.41 ± 0.22^a, b^
ApoB (g/L)	0.76 ± 0.15	0.73 ± 0.18	0.83 ± 0.21^a, b^	0.81 ± 0.20^a, b^
ApoB/apoA1	0.53 ± 0.13	0.52 ± 0.17	0.60 ± 0.18^a, b^	0.59 ± 0.18^a, b^
PAF-AH activity^d^				
Plasma PAF-AH (nmol/min/ml)	49.20 ± 10.27	47.20 ± 11.50	48.01 ± 12.50	46.39 ± 13.71
H-PAF-AH (nmol/min/ml)	5.12 ± 1.46	5.17 ± 1.59	4.61 ± 1.98^a, b^	4.76 ± 1.80
ApoB-PAF-AH (nmol/min/ml)	44.08 ± 9.40	42.03 ± 10.67	43.39 ± 11.49	41.63 ± 12.56
ApoB-PAF-AH/H-PAF-AH	9.07 ± 2.59	8.55 ± 2.29	10.42 ± 4.37^a, b^	9.33 ± 2.69^c^

Values are presented as the mean ± SD

Comparisons of all parameters were corrected for differences in age and BMI between the two subgroups except the parameters of age and BMI

^a^ *P* < 0.05, compared with *AA* genotype subgroup in controls

^b^*P* < 0.05, compared with *H1H2 + H2H2* genotype subgroup in controls

^c^*P* < 0.05, compared with *AA* genotype subgroup in PCOS patients

^d^Controls: *H1H1* (*n* = 165), *H1H2 + H2H2* (*n* = 97 + 15); PCOS: *H1H1* (*n* = 182), *H1H2 + H2H2* (*n* = 86 + 13)

Additionally, patients with the respective AA and AG + GG genotypes of the rs4420638 polymorphism or the respective *H1H1* and *H1H2+ H2H2* genotypes of the *-317H1/H2* polymorphism were more obese and had a higher F-G score, acne grade score and average ovarian volume, and more adverse hormonal, glucose and lipid metabolic profiles compared with the corresponding control subgroups. Patients with the *AA* or *H1H1* genotype had a decreased H-PAF-AH activity and an increased ratio of apoB-PAF-AH to H-PAF-AH compared with the corresponding *AA* or *H1H1* control subgroups (*P* < 0.05, Tables 4 and 5).

### *ApoC1 rs4420638A/G* and *-317H1/H2* allele status by *apoE ε2/ε3/ε4* alleles

The associations of *apoC1* allele status with *apoE ε2/ε3/ε4* allele status were observed in patients with PCOS and controls, as shown in Tables 6. A strong association of the *apoC1 H2* allele with the *apoE ε2* or *apoE ε4* allele and of the *apoC1 rs4420638 G* allele with the apo*ε4* allele was revealed in patients with PCOS and controls. Of the *apoE ε2* alleles evaluated, 273 (100%) were associated with *apoC1 H2* alleles. Of the 319 *apoE ε4* alleles, 303 (95%) were *H2* alleles or *rs4420638 G* alleles and only 16 (5%) were *H1* alleles or *rs4420638 A* alleles. In contrast, the *apoE ε3* allele was strongly associated with the *apoC1 H1 or rs4420638 A* alleles*.* Of the 2684 apo*ε3* alleles, 2604 (97%) were *H1* alleles and 2594 (96.6%) were *rs4420638 A* alleles; however, only 80 (3%) were *H2* alleles and 90 (3.4%) were *rs4420638 G* alleles. Nevertheless, these differences were not statistically significant for patients with PCOS versus the controls (*P* > 0.05, Table 6).

**Table 6:** *ApoC1/apoE* haplotypes in patients with PCOS and controls

*ApoE* controls/cases	*ApoC1–317*			*ApoC1 rs4420638*		
	*H1*	*H2*	*P*	*A*	*G*	*P*
*ε2*						
Controls	0	131 (100%)		131 (100%)	0	
PCOS	0	142 (100%)	–	139 (97.9%)	3 (2.1%)	0.094
Total	0	273 (100%)		270 (98.9%)	3 (1.1%)	
*ε3*						
Controls	1203 (96.7%)	41 (3.3%)		1194 (96.0%)	50 (4.0%)	
PCOS	1401 (97.3%)	39 (2.7%)	0.372	1400 (97.2%)	40 (2.8%)	0.075
Total	2604 (97.0%)	80 (3.0%)		2594 (96.6%)	90 (3.4%)	
*ε4*						
Controls	8 (5.4%)	139 (94.6%)		7 (4.8%)	140 (95.2%)	
PCOS	8 (4.7%)	164 (95.3%)	0.747	9 (5.2%)	163 (94.8%)	0.848
Total	16 (5.0%)	303 (95.0%)		16 (5.0%)	303 (95.0%)	

Haplotype data are presented as the number (%) of patients or controls

## Discussion

In this study, we show that *apoC1 rs4420638A/G* and *-317H1/H2* genetic polymorphisms are not associated with a risk of PCOS in Chinese women. However, compared with patients carrying the *AA* genotype, patients carrying the *G* allele (*AG* + *GG*) of the *rs4420638* polymorphism had significantly higher LH, TC, LDL-C, non-HDL-C and apoB levels, and a higher ratio of apoB to apoA1, and tended to have an increased ratio of LH to FSH. This suggests that this polymorphism may potentially be linked to endocrine abnormalities of the reproductive axis and dyslipidemia in the patients. In addition, we found a higher acne grade score and an increased ratio of apoB-PAF-AH to H-PAF-AH activity in patients carrying the *H1H1* genotype of the *-317H1/H2* polymorphism compared with patients carrying the *H2* allele. This suggests that the *-317H1/H2* genetic polymorphism is associated with systemic chronic inflammation in these patients. Furthermore, we demonstrated that the *rs4420638A/G* or *-317H1/H2* polymorphisms of the *apoC1* gene exist in moderate to reasonably high linkage disequilibrium with *apoE ε2/ε3/ε4* polymorphisms.

Several studies have found that the *rs4420638A/G* polymorphism in the *apoC1* gene is associated with serum lipid levels, chronic inflammation and disease risk. The *G* allele of rs4420638 is associated with a higher level of TC, LDL-C, TG and PAF-AH activity, abdominal obesity, and increased risk of type 2 diabetes and coronary heart disease [13–17]. On the other hand, the *G* allele is associated with lower serum C-reactive protein (CRP) levels [13]. In this study, we found that the *G* allele carriers were associated with not only increased serum LDL-C levels but also elevated serum non-HDL-C and apoB levels, and the ratio of apoB to apoA1 than the *AA* homozygotes in patients with PCOS or the controls. Because apoB and the apoB/apoA-I ratio have been reported to be better predictors of cardiovascular diseases than traditional lipid measurements [4, 33], our results provide further evidence that the *A → G* variant of rs4420638 in the *apoC1* gene is associated with cardiovascular disease risk. We also demonstrated that the controls with the *G* allele had higher fasting insulin and glucose concentrations, HOMA indexes, plasma PAF-AH and apoB-PAF-AH activity than the *AA* homozygotic controls. Additionally, patients carrying the *G* allele tended to have a reduced waist-to-hip ratio than the *AA* homozygotic patients. Consistent with our findings, Avery et al. demonstrated that the *rs4420638A/G* polymorphism of the apoC1 gene was associated with elevated plasma glucose, atherogenic dyslipidemia, vascular inflammation, and central obesity in individuals of European descent [34]. Grallert et al. reported that the *A → G* variant of *rs4420638* was associated with increased plasma PAF-AH activity [17]. Furthermore, in this study, we demonstrated that the *apoC1 rs4420638 G* allele exist in strong linkage disequilibrium with the *apoE ε4* allele, which may partially explain the effect of the *rs4420638 G* allele on lipoprotein metabolism and disease risk. The *apoE ε4* allele was associated not only with higher serum TC and LDL-C levels but also with higher oxidative stress, a more pro-inflammatory state and increased risk of cardiovascular disease and late-onset Alzheimer’s disease [30, 35]. For the first time, our study determined that the PCOS patients with the *G* allele had higher LH levels and tended to have increased ratio of LH to FSH than in patients with the *AA* genotype, suggesting that the *A → G* variant of rs4420638 may potentially link to endocrine abnormalities of the reproductive axis in the patients. However, the effect of this gene variant on the reproductive axis must be investigated further.

The *-317H1/H2* polymorphism of the *apoC1* gene, which is localized to the transcription initiation site in the promoter region, has been identified to exist in linkage disequilibrium with the *ε2/ε3/ε4* polymorphisms of the *apoE* gene and influences lipoprotein metabolism [19]. The *H2* allele of the *apoC1* gene has been reported to cause a significant 1.5-fold increase in apoC1 gene transcription in a reporter-gene assay [19]. The *H2* allele disrupts the binding of a transcription suppressor to produce a positive effect on transcription [19]. This allele also correlated with development of Alzheimer’s disease [20] and familial dysbetalipoproteinemia [21]. In this study, we found that compared with *H1H1* homozygotes, the *H2* allele was associated with lower ratio of apoB-PAF-AH to H-PAF-AH and acne grade score in patients with PCOS. Furthermore, the *H2* allele decreased or tended to decrease TC, LDL-C, non-HDL-C and apoB levels, but tended to increase waist-to-hip ratio and HOMA index in the controls. We also determined that the *apoC1 H2* allele has complete linkage disequilibrium with the *apoE ε2* allele, and to a lesser extent, the *apoE ε4* allele in Chinese women. Consistent with our findings, Xu et al. reported that the *H2* allele was associated with a decrease or tending decrease in apoB and LDL-C levels compared with *H1H1* homozygotes in African-Americans with the *apoE ε3/ε3* genotype but not the *apoE ε3/ε4* genotype [19]. Similar to apoE2, modest levels of apoC1 expression may decrease hepatic secretion of VLDL and apoB and produce lower LDL levels due to decreased partitioning of dietary lipids to the liver, relating to delayed remnant clearance, especially in the *apoE ε2* and *apoE ε3* contexts; however, higher apoC1 levels may increase plasma lipid levels via decreased remnant clearance [19]. Expression of apoC1 is upregulated and serum apoC1 levels are higher in patients with PCOS compared with the controls [23]. This may be one of the reasons why serum lipid profiles in the *apoC1–317H1/H2* polymorphism are different between patients and controls. Plasma PAF-AH specifically hydrolyzes and inactivates PAF and PAF-like oxidized phospholipids that are potent pro-inflammatory mediators [36]. Given that H-PAF-AH, an antioxidant enzyme of HDL, plays anti-inflammatory role and apoB-PAF-AH is associated with inflammation, it has recently been suggested that an increased ratio of apoB-PAF-AH to H-PAF-AH might be a marker for chronic inflammation [31, 32, 36, 37]. These results suggest that the *H1* allele might relate to increased systemic chronic inflammation in patients with PCOS.

In addition, our study also showed that the *A → G* variant and the *CGTT* insertion (*H2*) allele of *apoC1* appeared to have a greater impact on metabolic profile in the controls relative to in patients with PCOS (Tables 4 and 5). It have been demonstrated that in addition to genetic variations, other factors such as obesity, insulin resistance, etc., also have a significant impact on metabolic profile [4, 5, 38]. Since patients with PCOS are more obese and have a more severe insulin resistance compared with the controls in this study (Tables 1, 2, 4 and 5), it is possible that the effects of *apoC1* genetic variations on metabolic profile may be disturbed or weakened by these factors in patients. Further research is needed to clarify this issue.

In particular, the allele frequencies for *apoC1* and *apoE* genetic variants differ among ethnic groups, and these variants exhibit an ethnically distinct linkage disequilibrium pattern [19, 20, 30]. For example, the frequency of the *apoC1 H2* allele combined with *apoE ε2, ε3 and ε4* is 0.95, 0.02 and 0.85, respectively, in European-Americans; 1.00, 0.08 and 0.55, respectively, in African-Americans [19]; and 1.00, 0.03 and 0.95, respectively, in Chinese women. Therefore, it is possible that the relationships between the *apoC1* and *apoE* genetic polymorphisms and lipoprotein levels or diseases may differ among ethnic groups.

We should point out that this study has some limitations. Firstly, given the low frequency of homozygosity of minor alleles *rs4420638GG* and *-317H2H2*, we could not analyze them in the form of subgroups. A larger sample size of patients and controls are needed to properly evaluate dose-dependent genotype characteristics. Second, due to insufficient sampling, we did not measure plasma and lipoprotein-associated PAF-AH activity in some subjects, which might influence the statistical power of these parameters. Third, we could not determine plasma apoC1 concentrations due to plasma or serum sample inadequacy. Further study to detect apoC1 levels may help provide clues to the mechanisms responsible for the genetic association with PCOS.

## Conclusion

The present study demonstrates that *apoC1 rs4420638A/G* and *-317H1/H2* genetic polymorphisms are not associated with the risk of PCOS in Chinese women. However, the *apoC1 rs4420638A → G* variation may potentially be linked to elevated serum LH levels, a relatively high ratio of LH to FSH, and an adverse lipid metabolic profile; and the *-317 H1H1* genotype is associated with increased the ratio of apoB-PAF-AH to H-PAF-AH activity and the acne grade score in the patients with PCOS. Additionally, this study also indicates that *apoC1 rs4420638A/G* and *-317H1/H2* genetic variations are associated with an adverse glucose and lipid metabolic profile and the *rs4420638G* allele increases plasma PAF-AH and apoB-PAF-AH activity in the control women. These results suggest that *apoC1 rs4420638A/G* and *-317H1/H2* genetic variants are related to metabolic abnormalities and chronic inflammation in patients and controls, while the *rs4420638A/G* polymorphism might be involved in endocrine abnormalities of reproductive axis in patients.

## Abbreviations

AI: Atherogenic index
apoA1: Apolipoprotein A1
apoB: Apolipoprotein B
apoB-PAF-AH: apoB-containing lipoprotein-associated platelet activating factor acetylhydrolase
apoE: Apolipoprotein E
BMI: Body mass index
DBP: Diastolic blood pressure
E_2_: Estradiol
F-G score: Ferriman–Gallwey score
FSH: Follicle stimulating hormone
Glu: Glucose
HDL-C: High-density lipoprotein cholesterol
HOMA index: Homeostatic model assessment of insulin resistance
H-PAF-AH: HDL-associated PAF-AH
Ins: Insulin
LDL-C: Low-density lipoprotein cholesterol
LH: Luteinizing hormone
SBP: Systolic blood pressure
TC: Total cholesterol
TG: Triglycerides
TT: Total testosterone
